# Investigations on the synthesis and characterization of silver-doped MoO_3_ thin films for photocatalytic applications

**DOI:** 10.1038/s41598-024-84485-y

**Published:** 2025-01-06

**Authors:** Olfa Kamoun, Anis Akkari, Badriyah Alhalaili, Ruxandra Vidu, Najoua Turki-Kamoun

**Affiliations:** 1 Laboratoire de Physique de la Matière Condensée, Faculté des Sciences de Tunis, Université de Tunis El Manar, Tunis, 2092 Tunisia; 2 Nanotechnology and Advanced Materials Program, Kuwait Institute for Scientific Research, P.O. Box 24885, 13109 Safat, State of Kuwait; 3 Faculty of Materials Science and Engineering, University POLITEHNICA of Bucharest, 313 Splaiul Independentei, Bucharest, Romania; 4 American Romanian Academy of Arts and Sciences, P.O. Box 2761, Citrus Heights, CA 95611-2761 USA

**Keywords:** Silver, MoO_3_, Structural properties, Optical properties, Electrical properties, Photocatalysis, Dye degradation, Energy science and technology, Nanoscience and technology

## Abstract

In this study, we aimed to enhance the photocatalytic performance of molybdenum oxide (MoO_3_) thin films by doping with silver (Ag) via a spray pyrolysis technique. The primary objective for silver incorporation was intended to introduce additional energy levels into the band structure of MoO_3_, improving its efficiency. Structural, optical, and photocatalytic properties were analyzed using X-ray diffraction (XRD) and optical spectroscopy. XRD results confirmed an orthorhombic phase with a (040) preferential orientation for all samples. Optimal crystallinity was observed with 2% Ag doping, yielding an 84 nm crystallite size, while higher doping levels reduced crystallite size. Band gap energy narrowed from 3.07 eV (undoped) to 2.94 eV (2% Ag-doped), indicating electronic structure changes. Impedance spectroscopy revealed superior electrical properties at 4% Ag doping, enhancing charge transport. Photocatalytic performance, assessed via dye degradation, showed significant improvement with silver doping, the degradation rate peaking at 4% Ag. These results demonstrate that silver doping optimizes structural and electronic properties of MoO_3_ thin films, leading to enhanced photocatalytic activity.

## Introduction

Transition metal oxide semiconductor has garnered significant attention in recent years due to their technological applications, particularly in oxide photocatalyst and solar cells^1–5^. These solar cells represent a promising, low-cost photovoltaic technology thanks to their high-power conversion efficiency. Additionally, metal oxides have found extensive use in charge storage devices^6,7^, memory devices^8^, solid oxide fuel cells^9^ and supercapacitors^10^.

Molybdenum trioxide (MoO_3_) is a notable metal oxide with significant potential. As an n-type semiconductor with a wide band gap energy of around 3 eV^11^, MoO_3_ is a valuable component in photovoltaic cells. Its unique optical properties have led to extensive research, making it one of the most utilized materials in optical devices^12^ and smart windows^13^. Furthermore, MoO_3_ has diverse applications in chemical engineering, medicine, and agriculture^14,15^. Recently, due to its remarkable structural and electrical characteristics, MoO_3_ has been incorporated into transistors^16^, catalysts^17,18^, electrochemical capacitors^19^, gas sensors^20^, and the production of ceramics and glass^21^.

MoO_3_ exhibits three crystallographic structures: one stable phase, α-MoO_3_, and two metastable phases, β-MoO_3_ and h-MoO_3_^22^. The electrical properties of MoO_3_ and its derivatives are significantly influenced by the synthesis techniques and growth conditions^22^. Since 2011, MoO_3_ has been prepared using chemical techniques and deposited on substrates through various chemical and physical methods, including thermal evaporation^23^, spin coating^24^, chemical vapor deposition^25^, sol-gel processing^26^, hydrothermal methods^27^, and solid-state reactions^28^. However, most of these synthesis methods require high temperatures or pressures, extended processing times, and the use of closed systems with complex equipment.

MoO_3_ has a bandgap that efficiently absorbs visible light, making it highly suitable for photocatalytic applications^29^. Unlike copper oxide (Cu_2_O), which possesses a direct bandgap advantageous for visible light absorption but necessitates higher photon energies for electron excitation^30,31^. Additionally, MoO_3_ offers superior chemical stability and durability^32^.

Doping is a well-known method for modifying the properties of thin films semiconductor. Specifically, strategic doping can improve the physical characteristics of MoO_3_ thin films. Noble metals like gold (Au), silver (Ag), and platinum (Pt) are prime candidates among transition metals because they (i) capture photogenerated electrons, increasing the lifespan and mobility of these carriers, and (ii) display a surface plasmon resonance (SPR) effect when exposed to light^33,34^. Among these valuable metals, silver (Ag) is especially preferred in this study for its outstanding stability, photocatalytic properties, antimicrobial properties, photovoltaic features, and lower cost.

Doping of molybdenum trioxide with other metals has been explored to reduce its band gap energy and enhance various physical properties, depending on the nature and concentration of the dopants^35–40^. One notable application of these materials is their photocatalytic effect. Similar studies have been conducted on this material, where researchers examined the impact of doping with metals such as Dy^41^, Eu^42^, Ce^43^, Sn^44^ Au^45^ and Ag^7,15,44,46^ on their photocatalytic efficiency.

This doped MoO_3_ has shown enhanced photocatalytic activity, surpassing that of undoped materials. This improvement is attributed to the creation of an intermediate state associated with a reduced band gap, resulting in significantly increased degradation efficiency due to doping^47^.

The spray technique was chosen for this study^48,49^. Spray Pyrolysis, which originated in the 1980s, has evolved into a crucial technology for synthesizing thin film materials from precursor solutions^50^. Known for its adaptability and scalability in depositing uniform composite materials and diverse nanostructured functional materials, Spray Pyrolysis has been extensively applied over the past four decades in semiconductor thin films^50^. Its straightforward operation and high efficiency continue to drive research aimed at depositing thin films of noble metals, mineral oxides, metal oxides, chalcogenides, and superconducting materials using this method^50^. Notably, Spray Pyrolysis offers distinct advantages: it allows easy doping by adding dopants to the aqueous spray precursor solution, eliminates the need for expensive targets and high-quality substrates required by vacuum deposition methods, provides precise control over deposition rate and film thickness across a wide spray range, and operates at moderate temperatures (~ 200–500 °C) without restrictions on substrate dimensions or surface profiles^50^.

In this study, we detail the synthesis process of both pure MoO_3_ and Ag-doped MoO_3_ with varying Ag concentrations. We investigate the impact of these dopants on the structural and optical properties of MoO_3_. X-Ray Diffraction (XRD) was employed to examine the structure and phases present in the samples. Scanning Electron Microscopy (SEM) was utilized to study the sample morphology, while energy-dispersive X-ray analyses (EDAX) was used to analyze the chemical composition. UV-Vis Reflectance Spectroscopy (RS) measurements were conducted to analyze and measure the band gap. Additionally, the electrical properties were examined using Electrical Impedance Spectroscopy (EIS). The photocatalytic activity of nanosized MoO_3_, synthesized using spray pyrolysis, is evaluated for the degradation of model organic dyes like methylene blue (MB) and methyl orange (MO) in an aqueous medium. The degradation efficiency of MoO_3_ with varying levels of Ag doping is compared, and the results are thoroughly analyzed and discussed.

To our knowledge, there is currently no existing literature investigating the simplest spray deposited MoO_3_ for photocatalytic applications. This unique approach provides valuable insights into the potential of this stable and non-toxic material for achieving efficient and environmentally friendly photocatalysis.

## Experimental details

### Synthesis of Ag doped MoO_3_ thin films

Thin films were deposited on glass substrates by spray pyrolysis at 460 °C using the method described by Kamoun et al.^51^. The spray solution consists of 0.01 M aqueous solution of ammonium molybdate tetrahydrate [(NH_4_)_6_Mo_7_O_24_4H_2_O] and the silver source is AgCl. The Ag/Mo molar ratios were 0%, 2%, 4% and 6%. Compressed air was used as a carrier gas and was blown through a nozzle with a diameter of 0.5 mm at a pressure of 0.35 bar. The flow rate of the precursor mixture during deposition was 6.67·10^−5^ L/s. After deposition, the film was allowed to cool.

### Characterization of Ag doped MoO_3_ thin films

Crystal structure of the film was analyzed using a Philips PW 1729 X-ray machine Diffractometer with monochromatic Cu-Ka radiation (λ = 0.15405 nm). Optical reflectance R(λ) and transmittance T(λ) were measured in the wavelength range from 200 to 2000 nm using a Perkin-Elmer spectrophotometer. The electrical properties of undoped and Ag doped MoO_3_-Ag films were studied by electrical impedance spectroscopy at room temperature using an Agilent E4980A impedance analyzer in the frequency range [20 Hz–2 MHz].

Measurement of the photocatalytic decomposition of methylene blue (MB) was done using sunlight. Firstly, we place MoO_3_:Ag thin film sample with the size of ​​1 cm × 3 cm in 25 mL aqueous solution containing MB at a concentration of 10^−5^ mol/L, in dark, for about 30 min in order to establish the adsorption-desorption equilibrium, where the molecules of MB may be adsorbed on to the surface of sample. The photocatalytic activity of MoO_3_:Ag was evaluated against methylene blue dyes using a solar simulator with an intensity of 1000 W/m^2^, simulating natural sunlight AM (1.5) conditions.

## Results and discussion

### X-ray diffraction analysis

**Fig. 1:**
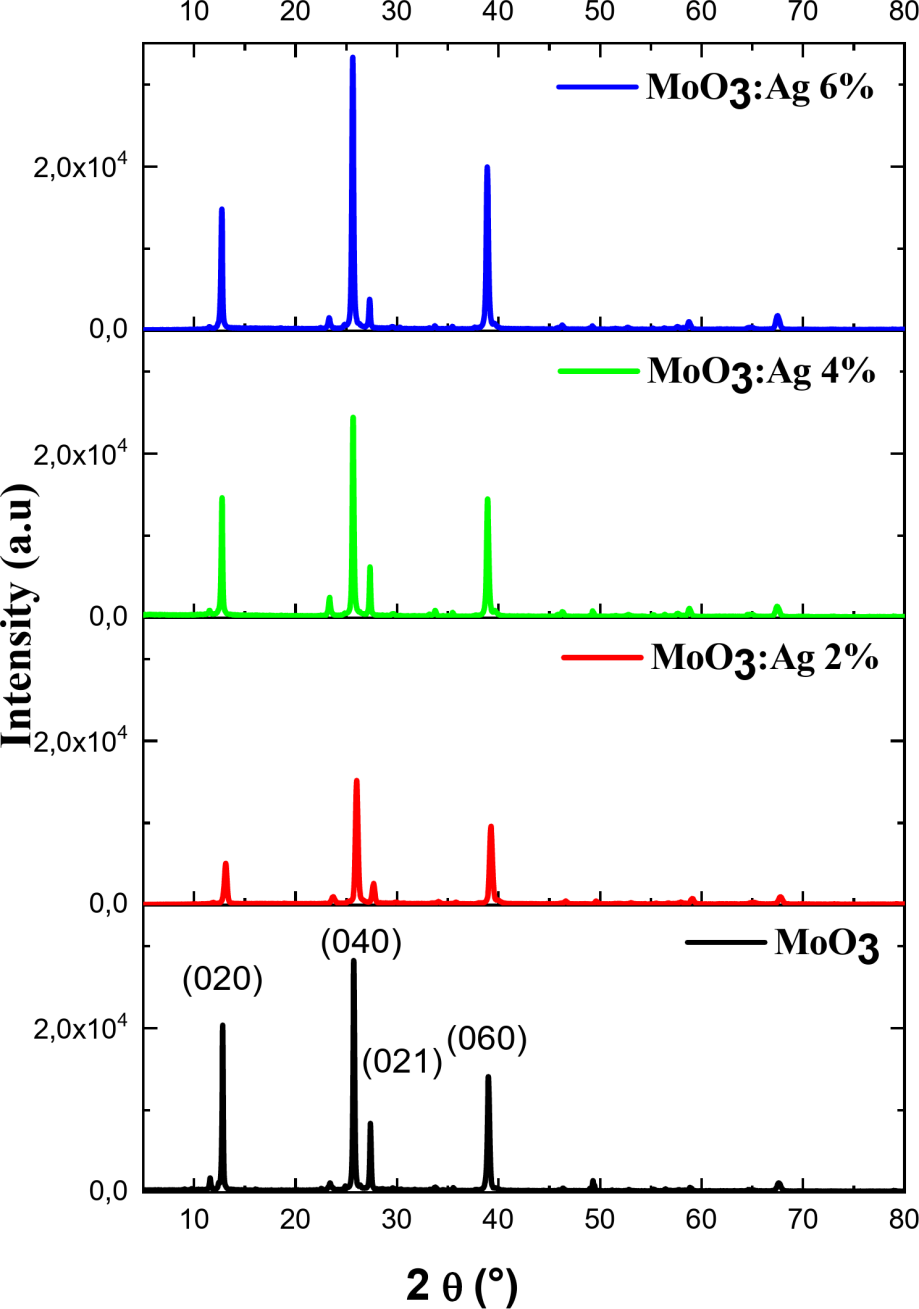
The XRD patterns of undoped and Ag doped MoO_3_ at different Ag content.

The XRD patterns of the undoped MoO_3_ films and Ag-doped MoO_3_ films with different molar concentrations are shown in Fig. 1. The XRD pattern shows that the MoO_3_ films are polycrystalline in nature, and the diffraction peaks obtained are relatively large.

MoO_3_ crystallizes in an orthorhombic structure, preferentially oriented on the (040) plane (standard JCPDS No. 05-0508^52^). The peaks are indexed to (0 2 0), (0 4 0) and (0 2 1), which is consistent with the orthorhombic system of the crystal phase α-MoO_3_. The strongest diffraction peak corresponds to the [0 4 0] direction.

The *k* values ​​of 2 and 4 in the (0 *k* 0) plane indicate that the film has good crystallinity and the MoO_3_ film was successfully deposited. Ag-doped MoO_3_ films exhibit diffraction peaks similar to those of crystalline MoO_3_. There are no other characteristic peaks corresponding to the Ag phase detected in the doped film, suggesting that Ag is not present as elemental phase but substitutes for Mo atoms in the crystal structure of MoO_3_. Song et al.^53^ suggested that Ag atoms doped uniformly in the MoO_3_ lattice without affecting the crystal structure of MoO_3_.

However, the peak position and intensity changed noticeably with Ag concentrations. Diffraction intensity reduction in MoO_3_:Ag (2%) is a well-known effect and usually occurs due to lattice strain caused by impurity atoms^54^. The peak intensity increases for MoO_3_:Ag (6%). The Ag^+^ ionic radius is 115 pm, which is larger than that of Mo^6+^ ions of 59 pm^53,55^. Furthermore, at high Ag/Mo doping ratios, a significant shift in the peak position towards a larger angle was observed compared to the undoped MoO_3_. The shift to higher angles is attributed to the reduction if the interlayer spacing by partial substitution of Mo atoms in MoO_3_ with Ag dopant atoms, confirming the incorporation of Ag ions into the MoO_3_ structure. Similar observations were reported by Munawar et al.^56^.

The interplanar spacing d_hkl_ values were calculated based on the principal phase by using Bragg equation:

1 $$2{d}_{hkl}\text{s}\text{i}\text{n}{\uptheta } = \text{n}{\uplambda }$$

where $n$ is the diffraction order (usually $n = 1$) and $\lambda$is the X-ray wavelength. The following relationship was used to determine the effect of different doping concentrations on the lattice parameters of MoO_3_:

2 $$\frac{1}{{d}_{hkl}^{2}}=\frac{{h}^{2}}{{a}^{2}}+\frac{{k}^{2}}{{b}^{2}}+\frac{{l}^{2}}{{c}^{2}}$$

where *a*,* b* and *c* are the lattice constants, $\left(hkl\right)$Miller index, and “$d$” is the distance between crystal planes. The estimated lattice constants are 3.93 Å, 13.66 Å, and 3.68 Å for *a*,* b*, and *c*, respectively, which agree well with standard JCPDS data for MoO_3_.

The Ag-doped MoO_3_ film has a preferential growth direction. Therefore, the lattice parameters such as grain size (D), microstrain (ε) and dislocation density (δ) are calculated using the Scherrer relationship as follows:

3 $$D=\frac{0.9 {\uplambda } }{ {\upbeta } \text{c}\text{o}\text{s}{\uptheta }}$$

4 $$\delta =\frac{1}{{D}^{2}}$$

5 $${\upvarepsilon }= \frac{{\upbeta }\text{c}\text{o}\text{s}{\uptheta }}{4}$$

where β is the FWHM, λ is the wavelength of Cu-Kα1 radiation (1.5406 Å) and θ is the Bragg diffraction angle. The calculated crystallite parameters are presented in Table 1.

**Table 1:** Crystallite size, dislocation density and lattice strain values of silver doped MoO_3_ thin films.

	D (nm)	δ $\times$10^14^ (lines/m^2^)	ε $\times$10^−4^
MoO_3_	94	1.13	3.67
MoO_3_:Ag 2%	84	1.41	4.12
MoO_3_:Ag 4%	80	1.56	4.33
MoO_3_:Ag 6%	58	2.99	5.99

By doping MoO_3_ with Ag, the average crystal size (D) gradually decreases. However, at a higher Ag doping level of 6%, the crystallite size decreases due to the higher dislocation density and microstrain of 2.99·10^14^ lines/m^2^ and 5.99·10^−4^, respectively.

### Optical properties

**Fig. 2:**
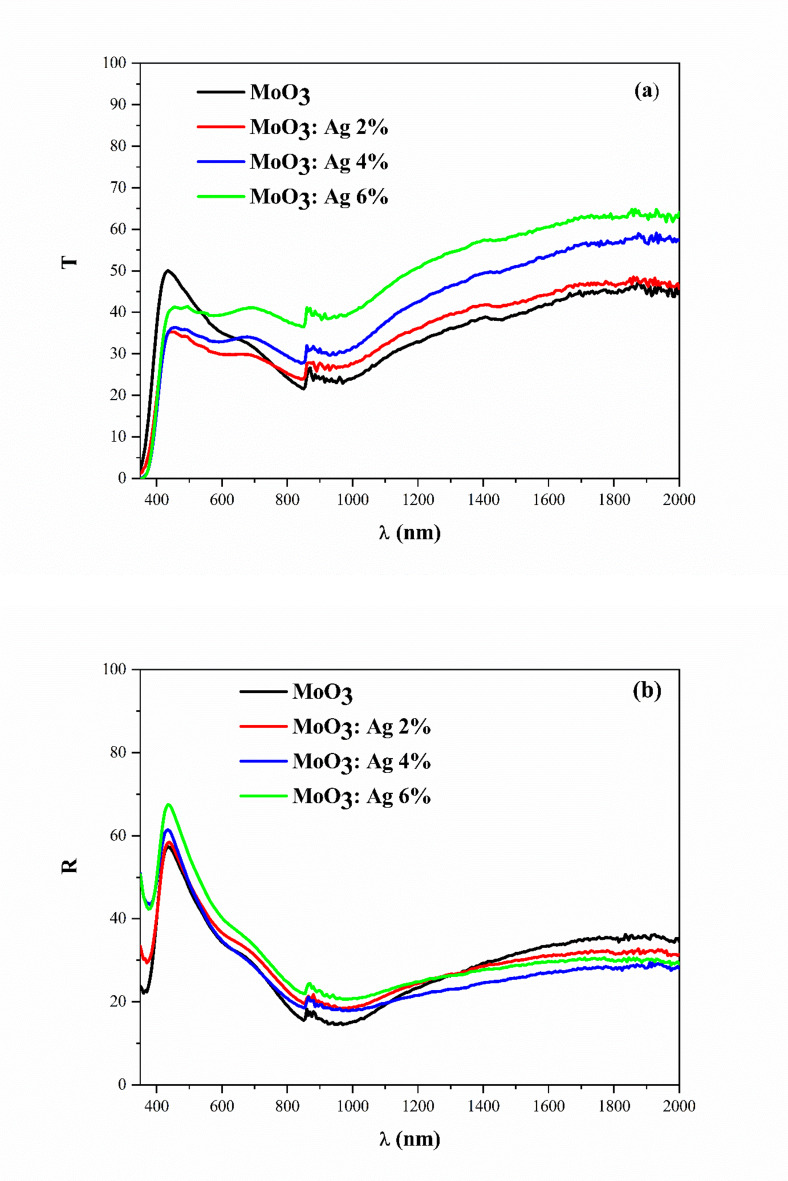
(**a**) Transmission and (**b**) reflectance spectra of undoped and Ag-doped MoO_3_.

The optical properties of the Ag-doped MoO_3_ thin films were examined using UV spectroscopy. The transmission and reflection measurements are shown in Fig. 2a and b, respectively. The average optical transmission is in the range of [30–60]% in the visible domain, while the reflectance in the visible range is in the range of [55–70]%. The Ag doping of MoO_3_ changes significantly in the transmission and reflection spectra. For the undoped MoO_3_ thin film, the highest transmittance, and the lowest reflectivity are obtained. The fundamental absorption edge of a semiconductor is the charge transfer barrier between the highest nearly full band and the lowest nearly empty band.

Regarding direct interband transitions, the intrinsic absorption edge of the film can be examined. The absorption coefficient (α) is determined using the relationship:

6 $$\alpha =\frac{4{\uppi }\text{k}}{{\uplambda }}$$

where *k* is the extinction coefficient and *λ* is the wavelength. A plot of (αhν)^n^ versus photon energy (*hν*), called a Tauc plot, is expected to show linear behavior in the higher energy region, corresponding to strong absorption around the absorption edge.

The optical absorption edge was analyzed by the following equation^57^:

7 $${\alpha h\nu =B(h\nu -Eg)}^{n}$$

where, *B* is a constant. *Eg* is the band-gap energy of the corresponding material, *n* is a number that has the values of 1/2 or 2 for direct transition and indirect ones, respectively.

**Fig. 3:**
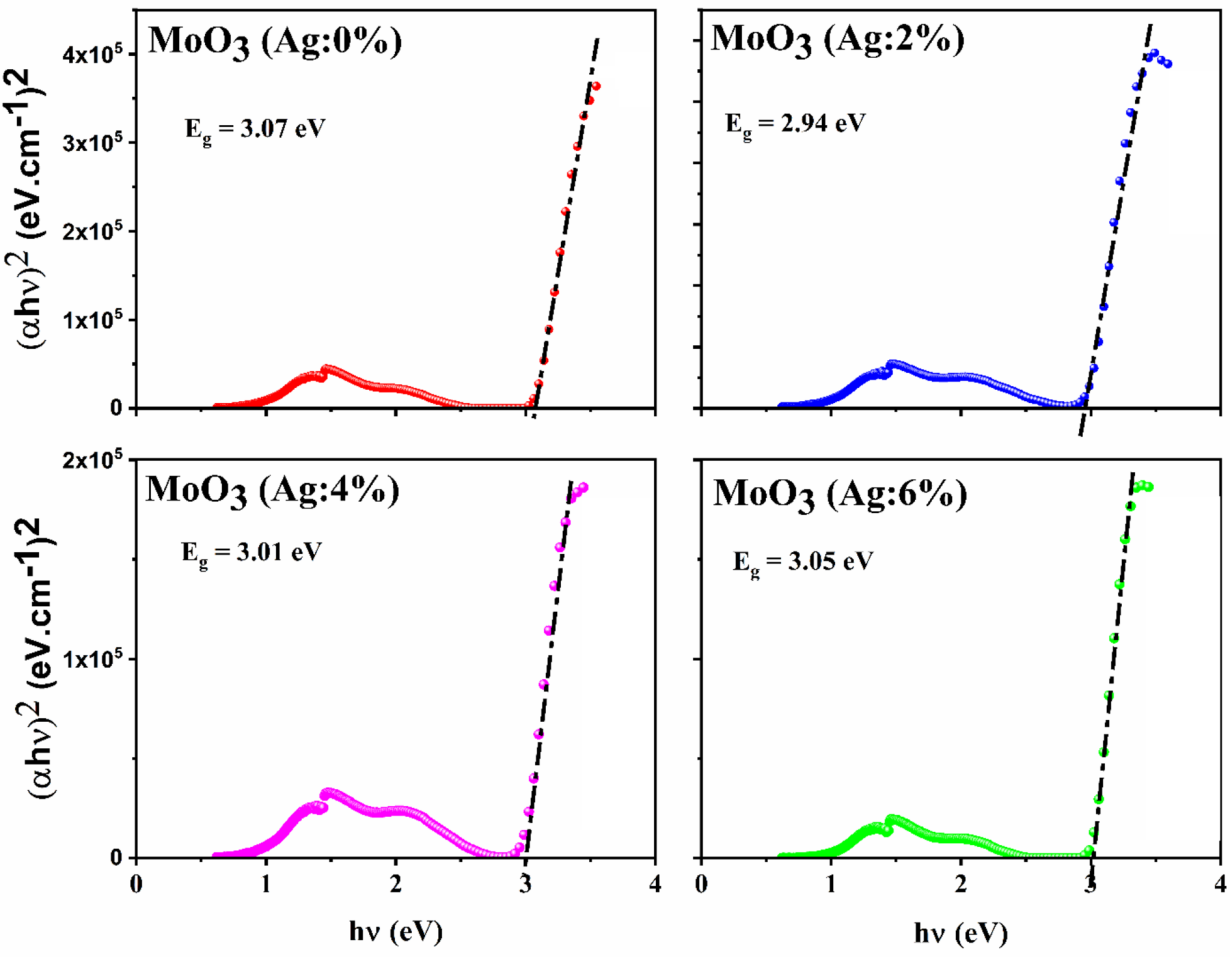
(αhν)^2^ vs. hν for MoO_3_:Ag thin films grown by spray for different Ag contents.

In Fig. 3, we report the variation of (*αhν*)^2^ as a function of *n*. Extrapolation of the linear component at (αhν)^2^ = 0 represents the band gap of MoO_3_:Ag material.

According to this figure, the bandgap value of pure MoO_3_ thin film was determined to be 3.07 eV, which decreased to approximately 2.94 eV for MoO_3_(Ag:2%). This result demonstrates that the introduction of Ag ions into the lattice creates additional energy levels in the band structure, resulting in a decrease in the bandgap value. This behavior was similar to that observed by other researchers^44,57^.

This is evident from the red shift of the shoulder in the transmission spectrum. This dopant is suitable for narrowing the band gap value by exploiting its valence state and sub-band states near the conduction band edge/maximum. However, this type of phenomenon can also be attributed to the creation of vacancies, defects, changes in crystallite size, and compositional changes of the dopants in the host lattice.

To study defects and their effects on optical transitions, the Urbach tail/energy was estimated. The coefficient of absorption follows the law presented in Eq. (8), where *α*_*0*_ is a constant and *E*_*U*_ is the Urbach energy, which characterizes the slope of the exponential limit:

8 $$\alpha ={\alpha }_{0}\, \text{exp}\left(\frac{h\nu }{{E}_{u}}\right)$$

**Fig. 4:**
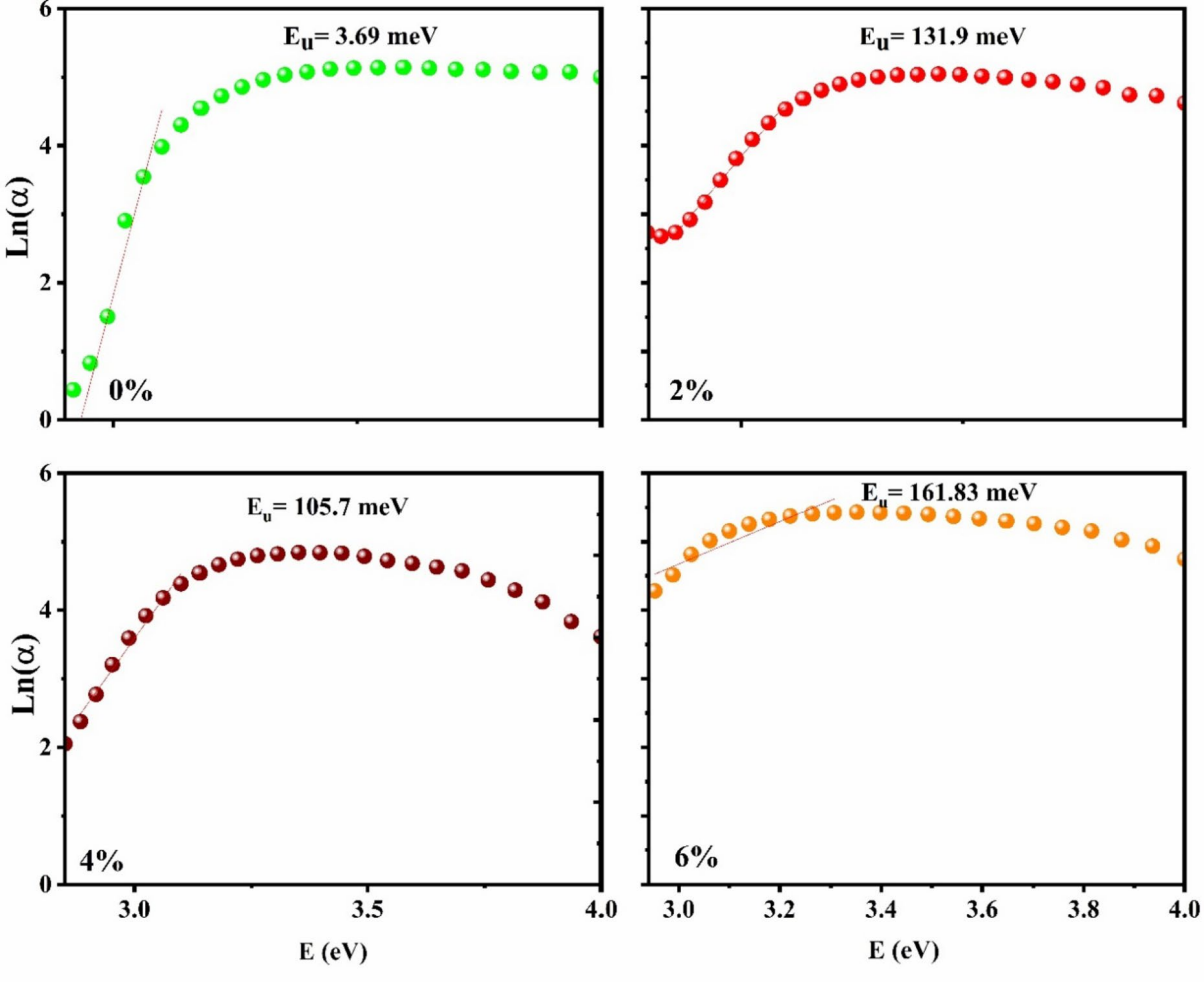
Ln(α) vs. *hν* spectra of MoO_3_:Ag thin film.

The logarithmic absorption coefficient curves versus the photon energy (eV) are plotted (see Fig. 4).

The slope is plotted in the linear region/absorption edge of the spectrum, and the reciprocal slope represents the Urbach energy (E_u_) of the system.

The observed trend indicates that the estimated E_u_ varies with the dopant levels up to MoO_3_:Ag 6%. The bare MoO_3_ thin-film structure exhibits an E_u_ of 3.69 meV, which increases to 161.9 meV upon incorporating Ag 6% into the host lattice. After that, E_u_ decreases with increasing Ag doping level, E_u_ equals to 131.9meV and 105.7 meV for Ag content equal to 2 and 4%, respectively.

All experiments were conducted at room temperature, where the thermal assistance to E_u_ can be deemed negligible. Consequently, the substitution of Ag at Mo-sites induces disorder in the electronic transitions and structural defects within the system. The random distribution of inserted Ag ions in the MoO_3_ lattice results in distinctions between the doped ions and the host Mo ions, causing disorder in the system. These changes may introduce sub-bands or new energy levels near the valence/conduction band, contributing to disorder or high E_u_ related to the doping concentrations.

### Electrical impedance spectroscopy study (EIS)

To understand how the presence of Ag doping affects the electronic properties of MoO_3_ films, electrical impedance measurements were performed at room temperature by applying an alternating current (AC) of 1 V and varying the frequency between 20 Hz and 2 Hz in dark, using an Agilent E4980 impedance analyzer. It is well known that EIS is a powerful technique that can study device interface properties^58^. In this study, we analyze the electrical properties of undoped and doped MoO_3_ films with different percentage of Ag concentrations (0%, 2%, 4%, and 6%). Impedance data can be visualized in two ways: by plotting the imaginary part (-Z”) versus the real part (Z’) (called a Nyquist plot) or by plotting amplitude (|Z|) and/or phase angle (ϕ) relative to log f (called Bode plot)^59^.

**Fig. 5:**
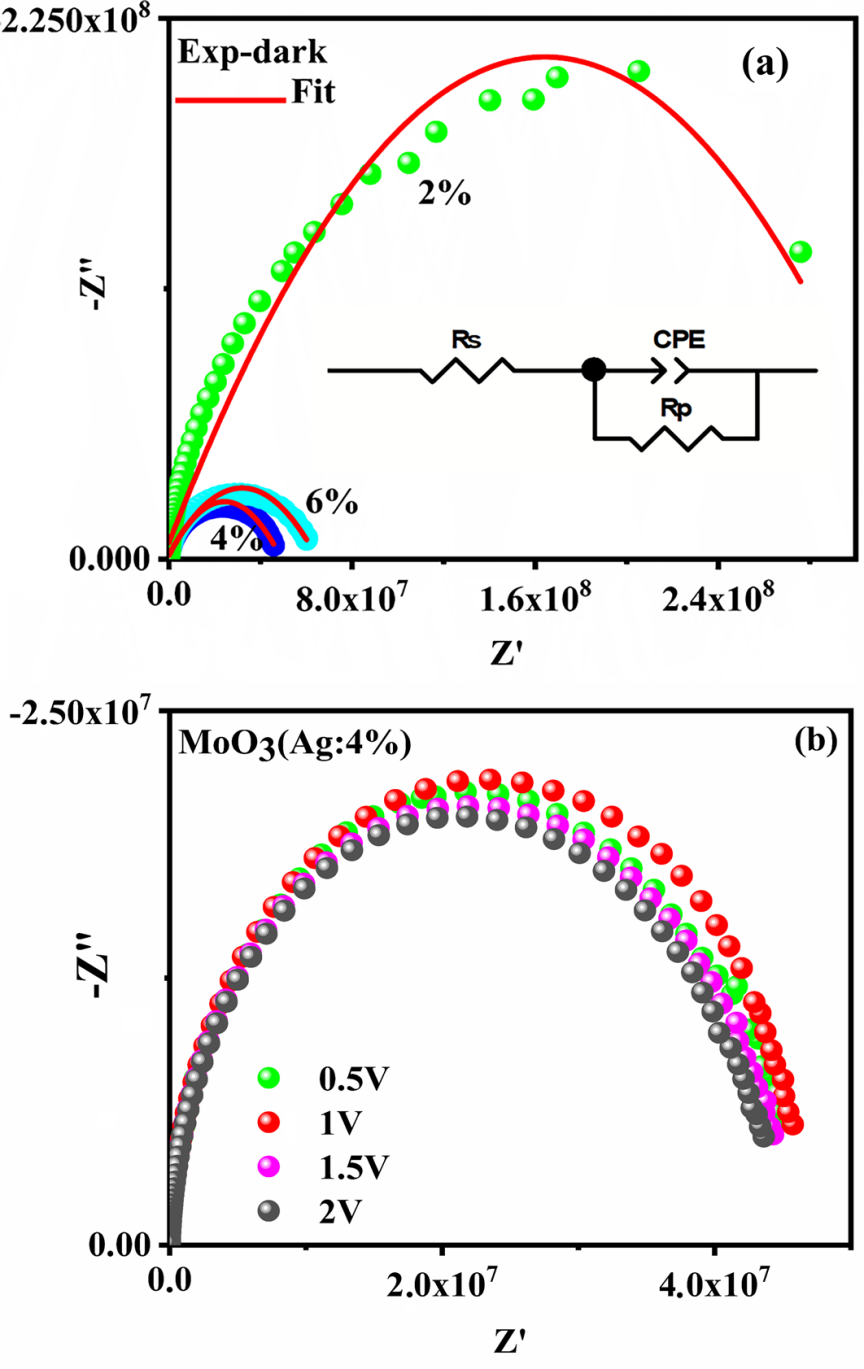
Nyquist plots of MoO_3_:Ag 4% thin films elaborated in dark.

Figure 5.a shows the Nyquist plot (-Z” vs. Z’) for all the films in the dark. As can be seen from this figure, the impedance spectrum of each film has a semicircular arc. Each film has a central semicircle, which is shifted toward the real axis, confirming the Debye relaxation process^60,61^. It can be seen from Fig. 5.a that the resistance of the MoO_3_ film is affected by silver doping concentration.

In particular, no silver doping produces a resistive layer, whereas for 4% Ag-doped MoO_3_ (Fig. 5b), a smallest semicircle with a diameter of approximately 4.7 × 10^7^ Ω is observed. This suggests that Ag improves charge transport between core particles through its electrical conductivity, thus reducing the overall impedance of the film. In fact, the results show that the MoO_3_ film doped with 4% Ag has good electrical properties.

In addition, the Nyquist plot derived from the experimental impedance spectrum data collected for the Ag-doped MoO_3_ films under different external bias voltages is shown in Fig. 5b. For all films, the impedance varied in the low-frequency region, indicating modulation of the grain boundaries by the external field. A similar trend was also observed for TiO_2_-G nanocomposite films^62^.

To analyze the respective contributions of different components of the material (grains, grain boundaries and electrodes), an equivalent circuit model is required^63^.For this purpose, the impedance curve is adjusted to an equivalent circuit using Zview software^63^. The equivalent circuit consists of a series resistor (Rs) and a constant phase element (CPE) in parallel with the resistor (Rp), as shown in the inset of Fig. 5.a. It was found that grains contribute at high frequencies, grain boundaries contribute at medium frequencies, and electrodes contribute at low frequencies^64^. Table 2 shows the equivalent circuit parameters of the Ag- MoO_3_ thin films determined by fitting the impedance data. Notably, the smallest resistance was observed for 4% Ag-doped MoO_3_. Similar behavior was observed by M. Layegh et al.^65^, for Co- MoO_3_ thin film.

**Table 2:** Parameter values for the equivalent electrical circuits shown in Fig. 5.

Sample	Rs (10^3^ Ω)	Rp (10^7^ Ω)	CPE-T (10^12^ F)	CPE-*P*(F)
MoO_3_	–	–	–	–
MoO_3_:Ag 2%	5.5	42.1	3.2	0.9
MoO_3_:Ag 4%	5.0	4.7	2.5	0.9
MoO_3_:Ag 6%	7.7	5.9	2.7	0.9

**Fig. 6:**
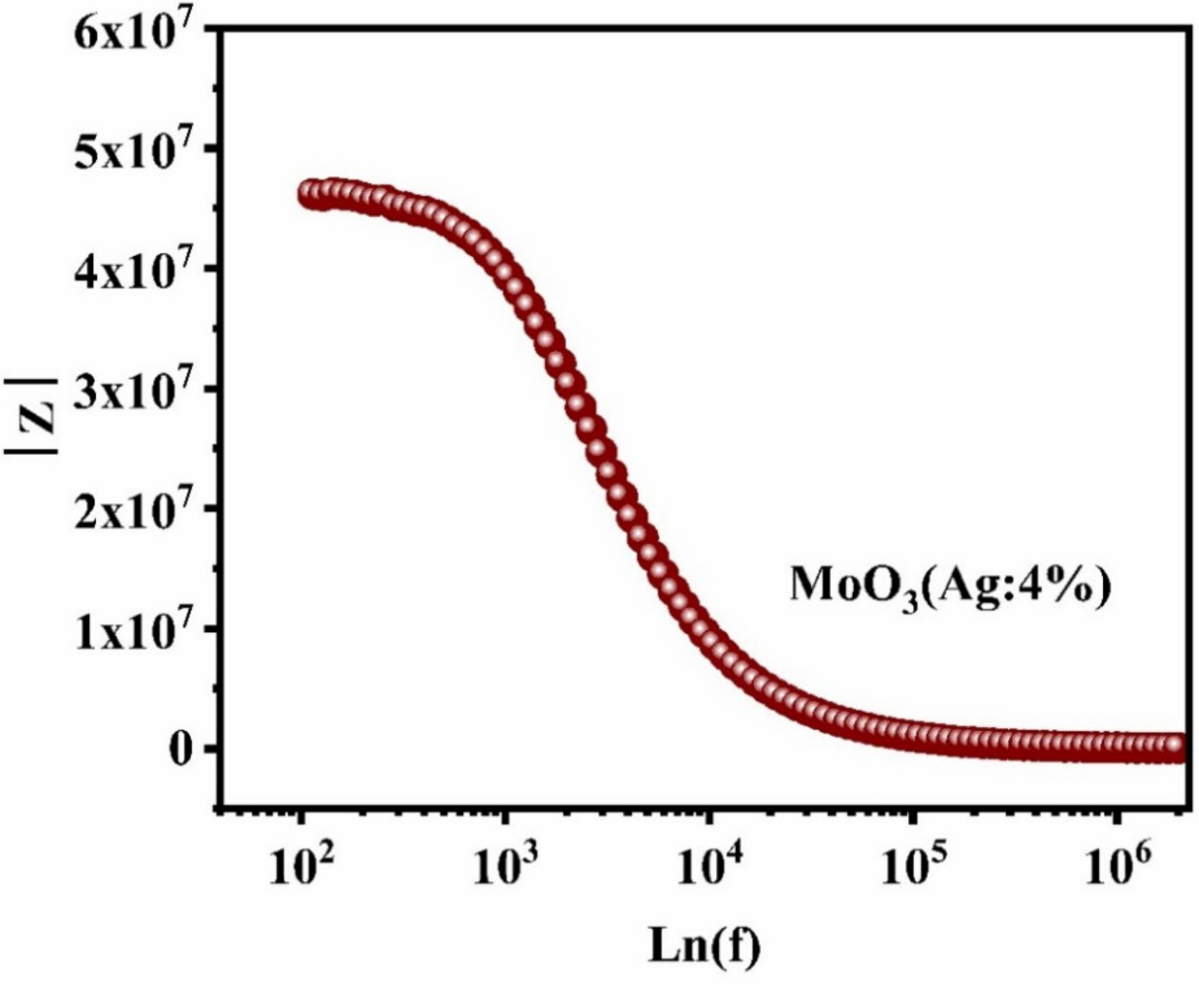
Impedance magnitude |Z| versus log f spectra of MoO_3_:Ag 4% thin films.

Figure 6 shows the magnitude of the impedance (|Z|) plotted against a semi-logarithmic frequency scale (log f). In the low frequency range (10^2^ Hz to 10^3^ Hz) the impedance represents the total resistance of the film^65^. Subsequently, the impedance amplitude decreases and reaches a minimum at high frequency (10^6^ Hz), indicating an increase in film conductivity^66^. Based on these results, we can conclude that adding silver can improve charge transport by changing the grain boundaries of MoO_3_ films. This makes Ag-MoO_3_ nanocomposite a promising candidate for optoelectronic applications.

### Scanning electron microscopy (SEM)

**Fig. 7:**
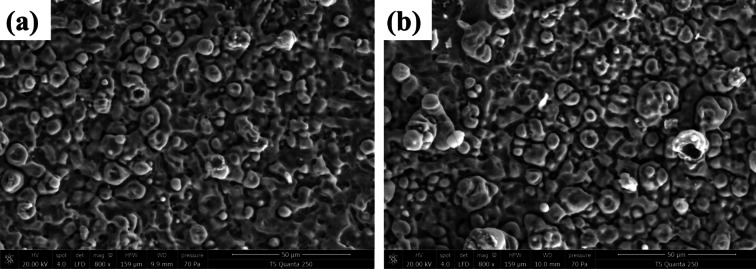
The SEM images of MoO_3_ undoped (**a**) and 4% silver-doped (**b**), synthesized via spray pyrolysis.

SEM analysis was conducted to study the morphology of undoped and doped MoO_3_:Ag 4% samples. Figure 7a,b presents the SEM images of undoped and 4% silver-doped MoO_3_, synthesized via spray pyrolysis. These images reveal features such as spherical particles, dark granular zones, fissures and crevices, and bright, shining regions, contributing to a diverse and complex surface. The 4% Ag-doped MoO3 exhibits a rougher, more uneven surface with pillar-like structures compared to the undoped sample, which enhances photocatalytic efficiency by increasing the specific surface area, reducing density, and improving permeability. This structural evolution highlights the potential for optimizing functionalities and uncovering novel physicochemical properties through silver doping.

**Fig. 8:**
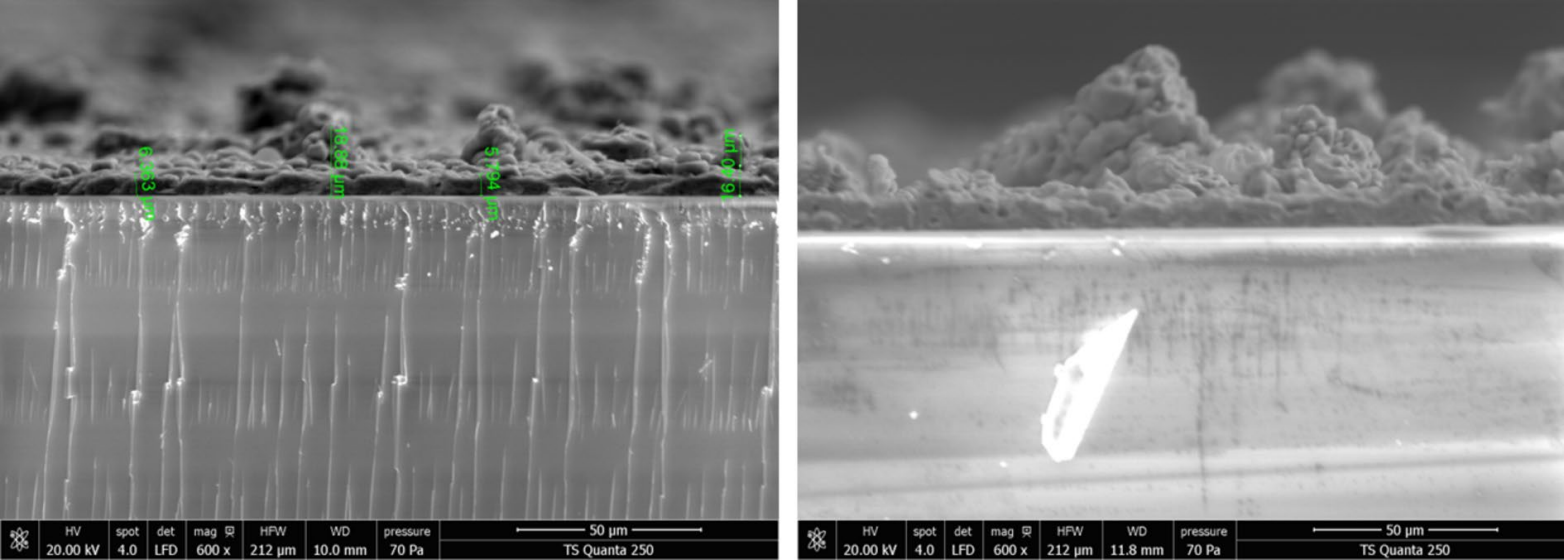
SEM cross section images of MoO_3_ undoped (**a**) and 4% silver-doped (**b**), synthesized via spray pyrolysis.

Figure 8a,b show SEM cross-sectional images of the undoped and 4% Ag-doped samples, respectively. The thickness of the MoO3 thin films increased from approximately 6 μm for the undoped sample to 9 μm after doping with 4% silver.

**Fig. 9:**
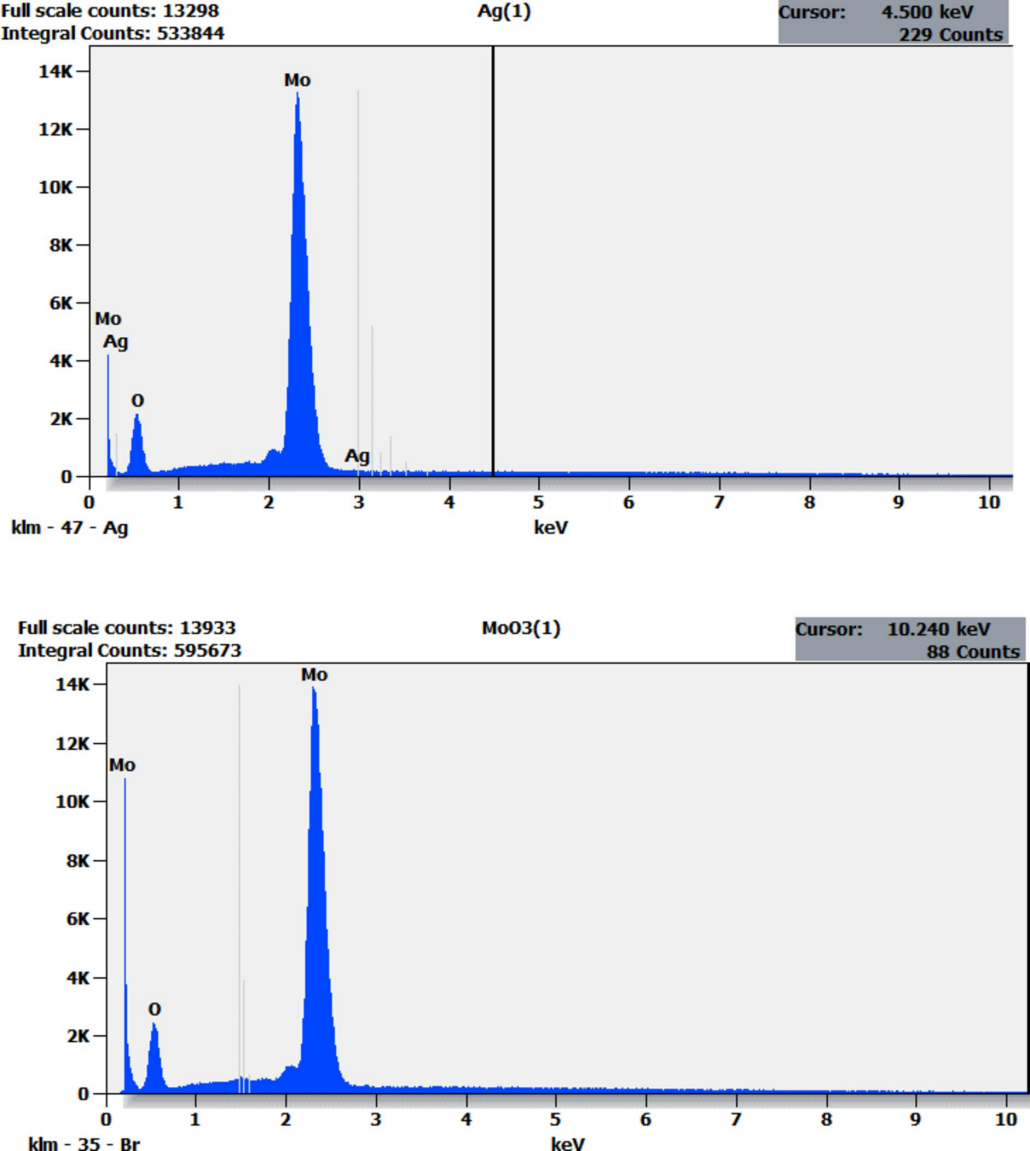
The EDX image of both Ag 4% doped (**a**) and undoped (**b**) MoO_3_.

Figure 9 presents the EDX image of both undoped and Ag 4% doped MoO_3_. The EDX spectra confirms the presence of Mo and O with an approximate atomic O-to-Mo ratio of 3.75, indicating the formation of MoO_3_. This ratio remains unchanged despite the increase of Ag doping, aligning with the findings of Khalid et al.^67^ and corroborated with XRD analysis.

### Photocatalytic study

We used the photocatalytic decomposition of methylene blue to test and compare the photocatalytic performance of undoped and Ag-doped MoO_3_. Before the photoreaction, dye adsorption with catalysts was performed for 30 min in dark conditions to achieve adsorption-desorption equilibrium.

**Fig. 10:**
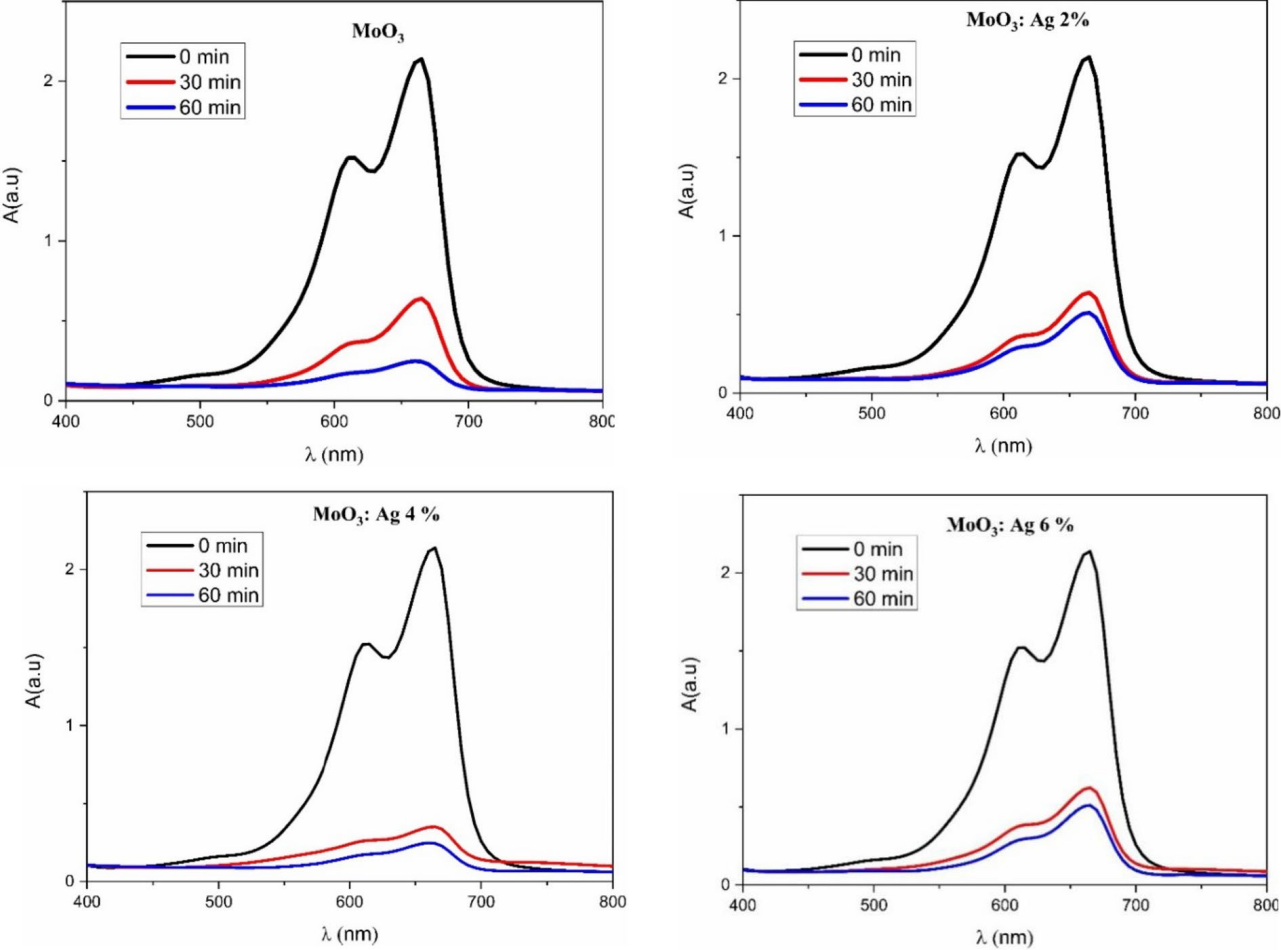
UV-Vis absorption spectra of MoO_3_:Ag thin films grown by spray pyrolysis.

Absorption spectrum of MB solution, without and with MoO_3_:Ag samples, are analyzed at different times. The UV-VIS absorption spectra of MB solution with MoO_3_:Ag thin films are shown in Fig. 10. There are two absorption peaks corresponding to methylene blue (MB) at 609 and 660 nm^51^.

Figure 10 shows that for both MoO_3_ and Ag-doped MoO_3_ the absorption decreases by increasing the irradiation time from 0 to 60 min. For MoO_3_:Ag, the peaks intensity decrease at Ag content from 2 to 4% and then increases for 6% Ag. The best MB degradation is obtained for MoO_3_:Ag 4% after 60 min of UV-VIS irradiation.

After 1 h of irradiation, approximately 98% of the MB amount was degraded by MoO_3_:Ag 4% (Fig. 10), while 70% was degraded in the presence of undoped MoO_3_ film.

**Fig. 11:**
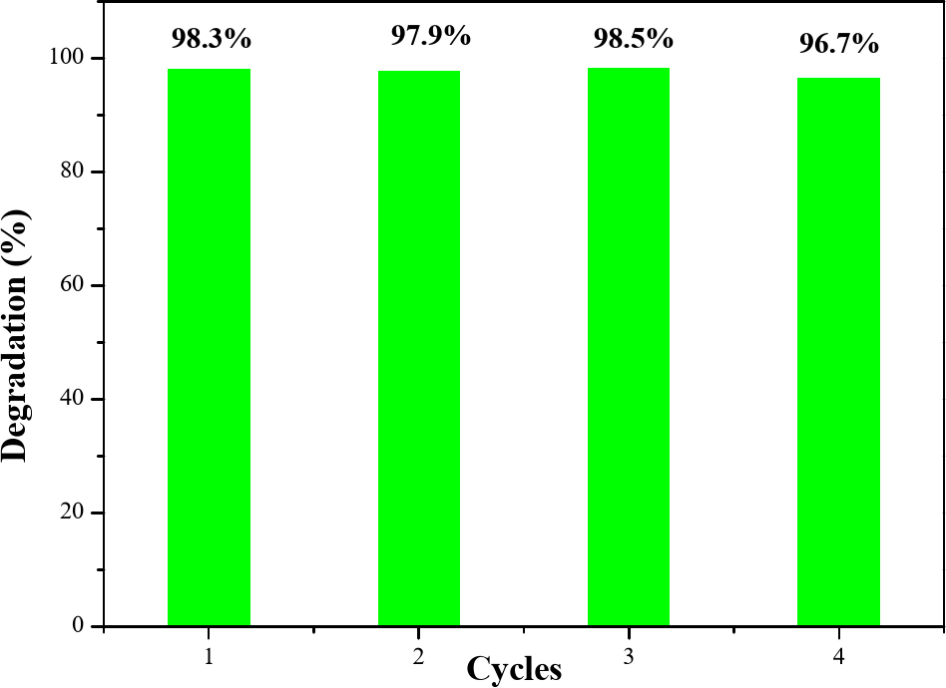
Reusability Silver Doped MoO_3_:Ag 4% for photocatalytic dye degradation.

To verify the reusability of the photocatalyst, MB degradation experiments were conducted for four cycles using MoO_3_:Ag 4% under diffused sunlight. As illustrated in Fig. 11, all four cycles showed degradation ranging from 96.7 to 98.5% during 60 min of irradiation, confirming that the MoO_3_:Ag 4% is quite stable and reusable.

### Photocatalytic activity

**Fig. 12:**
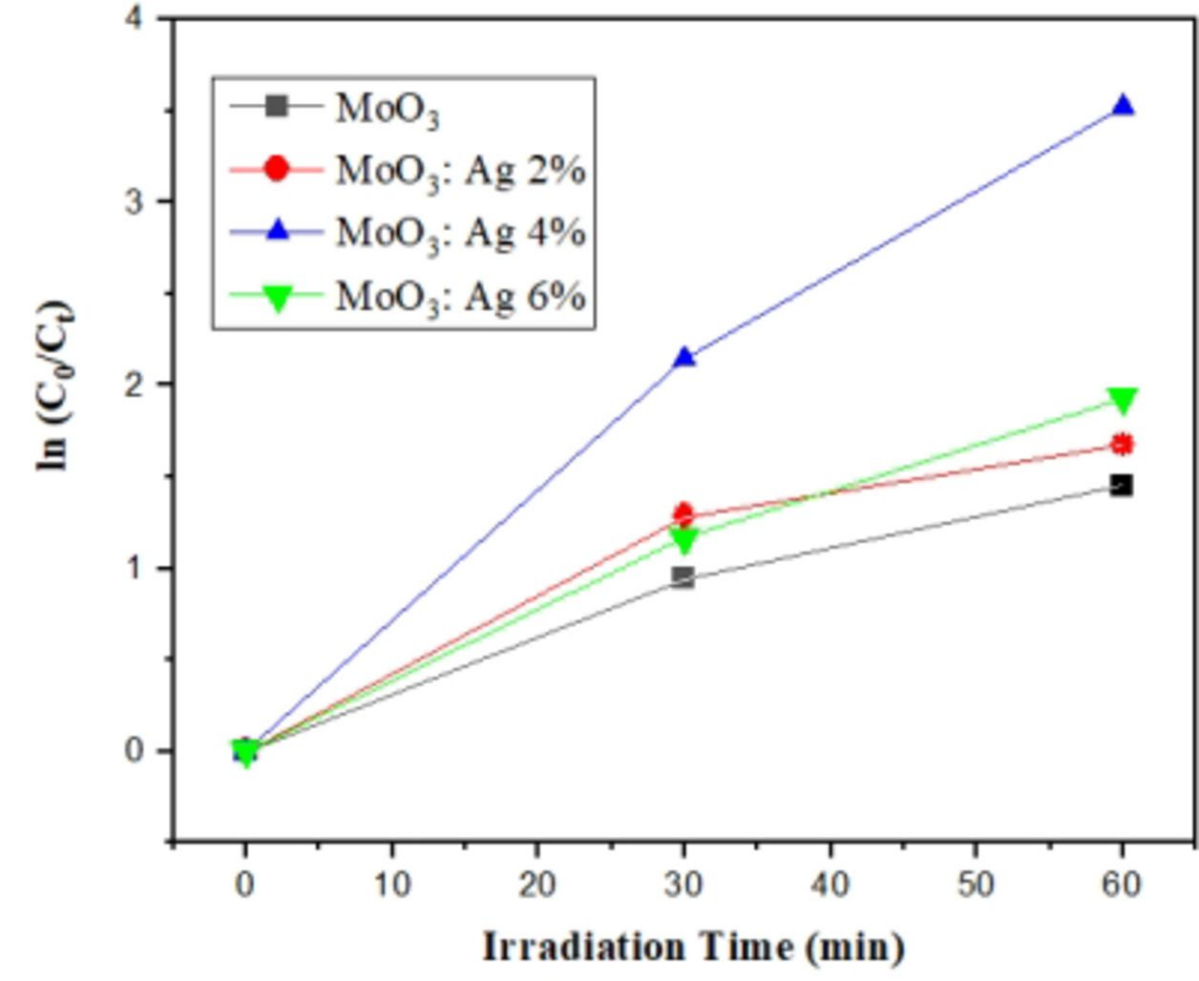
Degradation kinetics plots for MoO_3_ at different time intervals.

The photocatalytic activity was assessed through the degradation of methylene blue (MB) under sunlight irradiation. Figure 12 shows the photocatalytic degradation of MB with different thin films over time. The concentration of MB decreases significantly with Ag doping. The MoO_3_:Ag 4% showing the highest degradation rate. The degradation rate constants (k) were calculated using the first-order reaction model^4^ :

$$Ln\left({C}_{0}/{C}_{t}\right)=kt$$

Where C_0_, C_t_ and k are the MB concentration at t = 0, at t ≠ 0 and the degradation rate content respectively. The degradation rate constants are reported in Table 3.

**Table 3:** The degradation rate constants of MoO_3_:Ag thin films grown by spray pyrolysis.

Sample	Degradation rate constant (k) (min^−1^)
MoO_3_	0.0242
MoO_3_:Ag 2%	0.0279
MoO_3_:Ag 4%	0.0587
MoO_3_:Ag 6%	0.0321

## Discussion

The superior photocatalytic performance of MoO:Ag 4% is attributed to its improved conductivity (confirmed through IES measurements), surface alterations resulting from Ag doping, and adjusted bandgap (verified via Tauc plot).

The adjusted bandgap enhances the excitation of valence electrons. Furthermore, Ag doping serves as a blocking agent, potentially reducing the recombination of electron-hole pairs to enhance the catalytic activity of the photocatalyst^68,69^.

The presence of Ag in the photocatalyst suppressed the aggregation of MoO_3_, thereby improving charge transport towards the surface. The combined benefits of increased surface area, excellent light absorption properties, and enhanced electronic conductivity in the MoO_3_ sample synergistically enabled the degradation of nearly 98% of MB dye within just 1 h of solar irradiation. When exposed to light, the catalyst generates electron-hole pairs. The photoexcited electrons in the conduction band of MoO_3_ can react with water, reducing O_2_ to O^2–^. Concurrently, the photoinduced holes in the valence band of MoO_3_ can react with water to produce a substantial amount of ·OH radicals. The combination of ultrasound irradiation and diffused sunlight further boosts the production of ·OH radicals. These ·OH radicals are primarily responsible for the observed effective degradation of MB dye^70–72^.

Khalid et al.^67^ found that cobalt doping of MoO_3_ achieved 82.5% degradation of MB. On the other hand, Shanmugam Mahalingam et al. found that the G-αMoO_3_ catalyst achieved the highest degradation efficiencies of 97% and 96%^73^.

Furthermore, from the absorbance spectra of the MB dye solution during the photocatalytic reaction of MoO_3_ 4% (Fig. 10), it is evident that there is no shift observed in the maximum absorbance wavelength peaks of the dye at 663 nm and 615 nm in the visible light region. These absorbance bands disappear after the photocatalysis reaction. This disappearance indicates that the heterocyclic/benzene rings in the MB dye molecule have completely decomposed, resulting in the complete mineralization of MB.

It’s noteworthy to mention that discussing the SPR effect of small metal nanoparticles, particularly those 10 nm or smaller, presents challenges. Specifically, the silver particle sizes in the samples studied here were extremely small, approximately 0.5 nm, making it difficult to assess product selectivity based on exposed growth facets^74^.

The difference between photoactivities of these samples is possibly due to their different microstructures, as shown from AFM results. Similar behavior was observed by Navgire et al.^75^.

The photodegradation mechanism of silver doped MoO_3_ involves several key steps, including photon absorption, charge carrier separation, formation of reactive oxygen species (ROS), and degradation of organic pollutants. When exposed to light, the MoO_3_:Ag photocatalyst absorbs photons, promoting electrons from the valence band to the conduction band, creating electron-hole pairs. Silver acts as an electron trap, reducing the recombination rate of electrons and holes, increasing the availability of charge carriers for photocatalytic reactions.

Reactive oxygen species (ROS) are highly reactive and can attack organic pollutants, breaking down complex molecules into smaller, less harmful compounds, eventually leading to complete mineralization into CO₂ and H₂O. Silver nanoparticles aid in charge separation and have plasmonic properties that enhance light absorption, increasing the generation of electron-hole pairs and boosting photocatalytic efficiency. In summary, the photo degradation mechanism of silver doped MoO_3_ involves the absorption of light, generation and separation of electron-hole pairs, formation of reactive oxygen species, and degradation of organic pollutants^76^.

Table 3 clearly demonstrates that both the degradation rates are influenced by the film thickness as reveled by SEM. As anticipated, the data shows that degradation rates rise with increasing film thickness. The same observation was found by Yu et al.^77^.

Several studies have reported the effects of doping MoO_3_ with transition metals such as Fe, Co, Zn and Ag, rare earth elements like Eu, as well as with non-metals like C. This doping strategy aims to modify the physical properties of MoO_3_ and enhance catalysis, sensing, and electronic properties. The results are summarized in Table 4.

**Table 4:** Doping strategies for MoO_3_ with transition metals such as Fe, Co, Zn and Ag, rare earth elements (Eu), and non-metals (C) as reported in the literature.

Dopant	Synthesis method and structure	Effect on electrical conductivity	Anti-bacterial activity	Effect on optical properties	Morphology	Optical band gap	Crystal-lites size (nm)	Ref.
Fe	Hydrothermal Single orthorhombic phase	Significant increase	–	–	Unaffected	Decrease	Nanosheets 100–200	^57^
Co	Solid-state reaction Orthorhombic phases	–	–	–	Considerable impact	–	–	^78^
Zn	Hydrothermal Transition from α to β orthorhombic phases	–	–	Improvement in photocatalytic properties	Unaffected	Decrease	45	^22^
C	Impregnation method Orthorhombic phases	–	–	Improvement in photocatalytic properties	Considerable impact	–	$\sim12$	^75^
Eu	Spray Orthorhombic phases	–	–	Improvement in photocatalytic properties	Affected	Decrease	75	^51^
Ag	Hydrothermal Metastable hexagonal phase	Increase	Increase	Improvement in photocatalytic properties	Considerable impact	Decrease	$\sim21$	^15^
	Self-assembly route method Metastable hexagonal phase	–	–	Improvement in photocatalytic properties (98.8%) Stable performance	Unaffected	Decrease	15.7	^71^
	Spray Orthorhombic phases	Increase	–	Improvement in photocatalytic properties (98%) Stable performance	Unaffected	Decrease	80	Our results

Our study demonstrates that Ag doping uniquely increases both the electrical conductivity and optical properties of MoO_3_, which can be particularly beneficial for applications in optoelectronic devices. However, Ag doping may be limited by higher costs and lower availability compared to other dopants such as Fe or Co. Therefore, while Ag doping offers distinct advantages, it is crucial to consider the specific application requirements to select the most appropriate dopant. Finally, further studies are in progress to test these films in optoelectronic applications.

## Conclusion

The spray synthesis of silver-doped molybdenum oxide (MoO_3_:Ag) was successfully demonstrated for the first time. Remarkable performances were obtained, especially in the field of photocatalysis under sunlight within 60 min. This innovative approach highlights the potential of MoO_3_:Ag as a highly effective photocatalytic material for solar-driven applications.

The simplicity of the spray method used to fabricate MoO3 thin films is particularly notable, offering a practical route to creating materials with significant specific surface areas. These properties, combined with the potential for doping with various transition elements, make MoO_3_ thin films highly versatile for applications in a wide range of sensitive devices including solar cells, gas sensors, and biosensors.

## Data Availability

All data generated or analyzed during this study are included in this published article.
